# Comparison of esketamine versus dexmedetomidine for attenuation of cardiovascular stress response to double-lumen tracheal tube intubation: a randomized controlled trial

**DOI:** 10.3389/fcvm.2023.1289841

**Published:** 2023-12-21

**Authors:** Chunyu Liu, Tianhua Zhang, Longhui Cao, Wenqian Lin

**Affiliations:** ^1^ Department of Anesthesiology, State Key Laboratory of Oncology in South China and Collaborative Innovation Center for Cancer Medicine, Guangdong Esophageal Cancer Institute, Sun Yat-Sen University Cancer Center, Guangzhou, China; ^2^ Department of Anesthesiology, Chongqing University Cancer Hospital, Chongqing, China; ^3^ Department of Blood Transfusion, State Key Laboratory of Oncology in South China and Collaborative Innovation Center for Cancer Medicine, Guangdong Esophageal Cancer Institute, Sun Yat-Sen University Cancer Center, Guangzhou, China

**Keywords:** dexmedetomidine, esketamine, intubation, laryngoscopy, double lumen tube

## Abstract

**Introduction:**

The insertion of a double-lumen tracheal tube may cause a transient but more intense sympathetic response. We examined the effects of esketamine vs. dexmedetomidine as an adjuvant to anesthesia induction to blunt double lumen tracheal (DLT) intubation induced cardiovascular stress response.

**Methods:**

In a randomized, double-blind trial, 78 adult patients scheduled for elective thoracotomy under general anesthesia requiring DLT intubation were enrolled. The patients were randomly divided into three groups: each group received one of the following drugs prior to induction of anesthesia: dexmedetomidine 0.8 µg/kg (Group A), esketamine 0.5 mg/kg (Group B), or normal saline (group C). The primary outcome was the incidence of a DLT intubation-related cardiovascular stress response, defined as an increase in mean arterial pressure or heart rate of >30% above the baseline values. The secondary outcomes were changes in hemodynamic and cardiac function.

**Results:**

The incidence of the response to cardiovascular stress was 23.1%, 30.8%, and 65.4% in groups A, B, and C, respectively. There was a significant decrease in intubation response in groups A and B in comparison with group C (*P* < 0.01); however, there was no significant difference between group A and group B (*P* > 0.05). Following the drug infusion and the induction of anesthesia, there was a significant decrease in HR and cardiac output in group A compared with group B. In contrast, no significant differences were observed in the left ventricular ejection fraction or in stroke volume between the three groups during induction of anesthesia.

**Discussion:**

Esketamine 0.5 mg/kg and dexmedetomidine 0.8 µg/kg attenuate cardiovascular stress responses related to DLT intubation. As adjuvants to etomidate induction, they do not impair cardiac function (ChiCTR1900028030).

## 1. Introduction

The insertion of the double-lumen tracheal tube (DLT) is the lung isolation technique of choice for thoracic surgery (1). Compared with the single-lumen tracheal tube (SLT), the DLT has a more rigid structure and a larger external diameter and needs to be inserted into the bronchi, which can evoke a transient but more intense sympathetic response manifesting as increased heart rate, blood pressure, and changes in ischemic ST-segment during placement (2). These responses to cardiovascular stress may be fatal in susceptible patients such as those with coronary artery disease, hypertension, and intracranial aneurysm (3).

Deepening the anesthesia and using drugs such as dexmedetomidine pretreatment is a commonly used approach to blunting the adverse cardiovascular stress response to intubation (3). However, previous studies have reported that this is often accompanied by a period of cardiovascular instability during anesthesia induction, especially when a combination of propofol with dexmedetomidine is used, and is characterized by hypotension and bradycardia before laryngoscopy and intubation, followed by hypertension and tachycardia (4). Such variation in cardiovascular status may change the fine balance between myocardial oxygen demand and supply, thus accelerating myocardial ischemia (5, 6). Moreover, as many thoracic patients who require DLT intubation are among the older population, and many are cancer patients who have received chemotherapy or radiotherapy before surgery. This increases their sensitivity, allowing less tolerance to cardiovascular changes (7). Therefore, preserving the hemodynamic stability during anesthesia induction and ultimately attenuating DLT intubation-related cardiovascular stress response is critical to this group of patients.

Esketamine, a new derivative of ketamine, is a non-selective, non-competitive antagonist of the NMDA receptor with sympathomimetic effect, cardiac stimulatory properties, analgesic and sedative activities (8). Previous studies using a combination of propofol with esketamine for sedation have shown a reduction in the dosage of individual drugs, a more stable hemodynamic response, and an attenuated stress response (9). However, little is known about the effects of esketamine on the intubation response.

Etomidate, a commonly used anesthetic agent with minimal depressant effects on the cardiovascular system, is recommended for induction of anesthesia induction in older adult patients (10). However, there are few studies evaluating dexmedetomidine or esketamine as an adjuvant to induction of etomidate on hemodynamic change and responses to cardiovascular stress especially with intubation for DLT. Thus, the aim of this study is to use transthoracic echocardiography (TTE) to further investigate esketamine or dexmedetomidine and its direct cardiac effect. We hypothesized that esketamine and dexmedetomidine can decrease the response to cardiovascular stress related to DLT intubation without affecting cardiac function.

## 2. Materials and methods

This study was approved by the Ethics Committee of the Sun Yat-Sen University Cancer Center (B2020-370-01) and written informed consent was obtained from all subjects participating in the trial. The trial was registered prior to patient enrollment at Chinese Clinical Trial Registry (Clinical trial number: ChiCTR1900028030, registry URL: https://www.chictr.org.cn/showproj.html?proj=46652).

### 2.1. Patients

**Figure 1:**
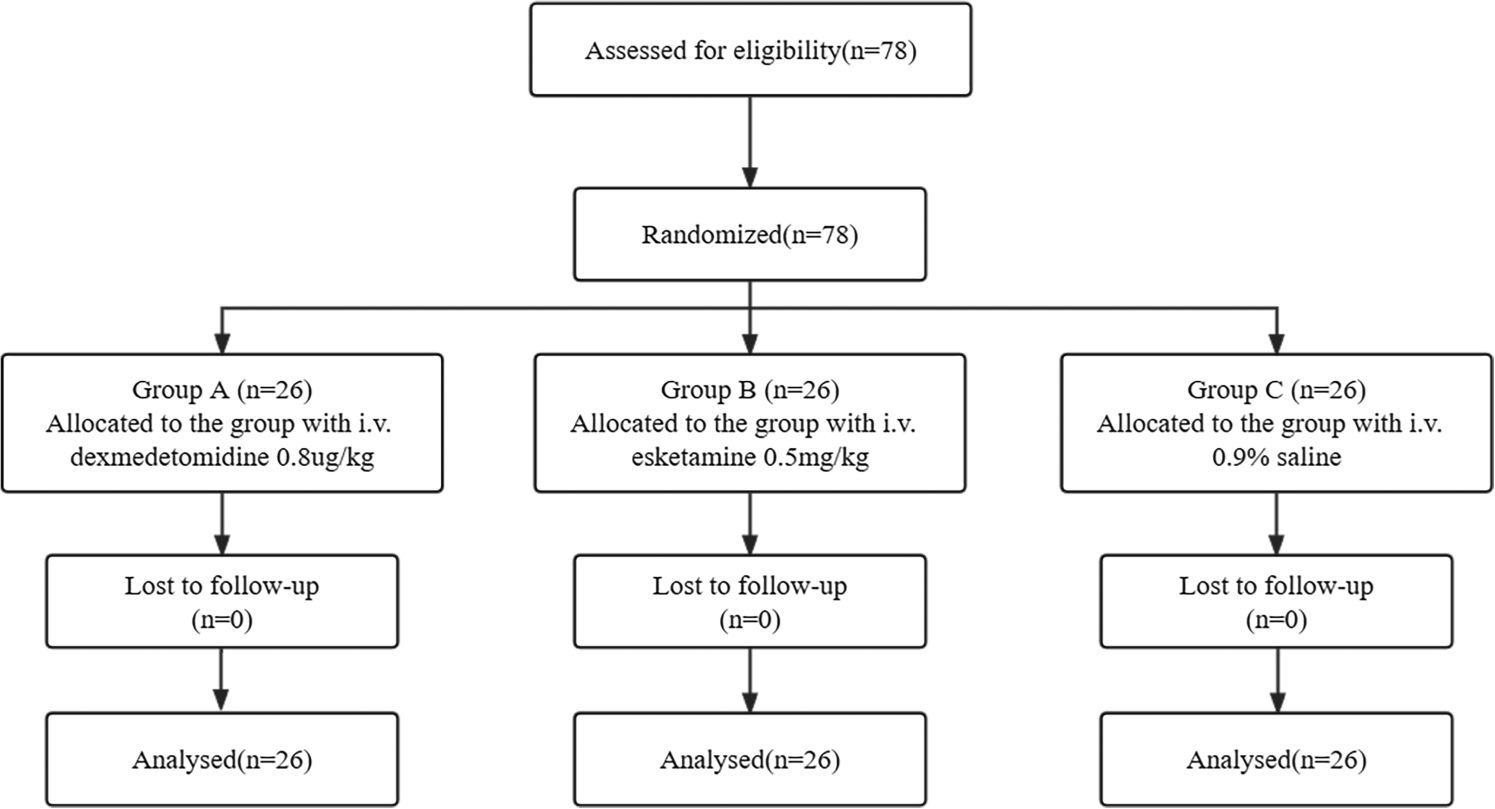
Consolidating standards of reporting trials (CONSORT) diagram.

Inclusion criteria were patients with American Society of Anesthesiologists Physical Status I to II, aged 20–75 years, scheduled for elective thoracotomy under general anesthesia requiring DLT intubation during the period from January 2021 to June 2021 (Figure 1).

The exclusion criteria included allergy to any of the drugs used, uncontrolled hypertension, ischemic heart disease, and renal or hepatic dysfunction. Those with anticipated difficult airways or requiring more than one attempt were also not included in the study.

After the patients arrived in the operating room and had a quiet rest for 10 min, monitoring was performed by pulse oximetry, electrocardiography, non-invasive blood pressure, and the Narcotrend® Index (NI) was determined. An intravenous line was established using a 20/18G cannula and Ringer lactate infusion was started at 10 ml/kg/h. Baseline hemodynamic parameters, such as heart rate (HR), systolic blood pressure (SBP), and mean arterial pressure (mAP), were recorded. Rate pressure products (RPP) were also recorded, which were calculated from SBP and HR recordings (formula: SBP × HR) and is an index of myocardial oxygen demand (11). TTE was performed by an anesthetist using two-dimensional echocardiography in M mode and obtained standard views of the apical four chambers, the apical two chambers, and the parasternal long axis. Ultrasonography was used to evaluate the end diastolic volume of the left ventricle (LVEDV), the end systolic volume of the left ventricle (LVESV), and the stroke volume (SV) (12). Cardiac output (CO) was calculated by SV multiplied by HR (formula: SV × HR). The left ventricular ejection fraction (LVEF) was calculated using SV divided by LVEDV (formula: SV/LVEDV). All variables were means of values measured over three cardiac cycles at end-expiration.

### 2.2. Randomization and intervention

Randomization was performed using a computer-generated random number list. Group assignment numbers were sealed in an envelope and kept by the study supervisor. After the baseline values were recorded, the opaque envelope was unsealed to determine which drug would be used.

The patients were randomly allocated into one of the three groups according to the drug used. Group A received dexmedetomidine 0.8 µg/kg, group B received esketamine 0.5 mg/kg based on our pilot study and a previous study (13), and group C received normal saline. These drugs were prepared by a blinded anesthetist, diluted in 20 ml of normal saline, infused for 10 min with a pump, and started before induction of anesthesia.

### 2.3. Anesthesia protocol

After 5 min of tested drug infusion, anesthesia was then induced using intravenous sufentanil (0.3 µg/kg) followed by etomidate (0.2–0.3 mg/kg) titrated to the loss of eyelash response guided by the Narcotrend® Index (NI). Cisatracurium (0.3 mg/kg) was administered to achieve neuromuscular relaxation and facilitate DLT intubation. All intubations were attempted 5 min after injectin the cisatracurium according to routine clinical practice by the same anesthetist. No surgical or any other stimulus was applied during the study period. Intraoperatively, anesthesia was maintained with sevoflurane 0.8 – 1.2 MAC in oxygen, remifentanil 0.1 μg/kg/min, and cisatracurium 2 μg/kg/min. Atropine was administered when the heart rate decreased to <45 beats.min^−1^. When mean arterial pressure decreased to below 70% of baseline value, ephedrine was administered. At the end of the surgery, a residual neuromuscular blockade was reversed with neostigmine 0.05 mg/kg and atropine 0.01 mg/kg used intravenously.

### 2.4. Outcome measures

**Figure 2:**
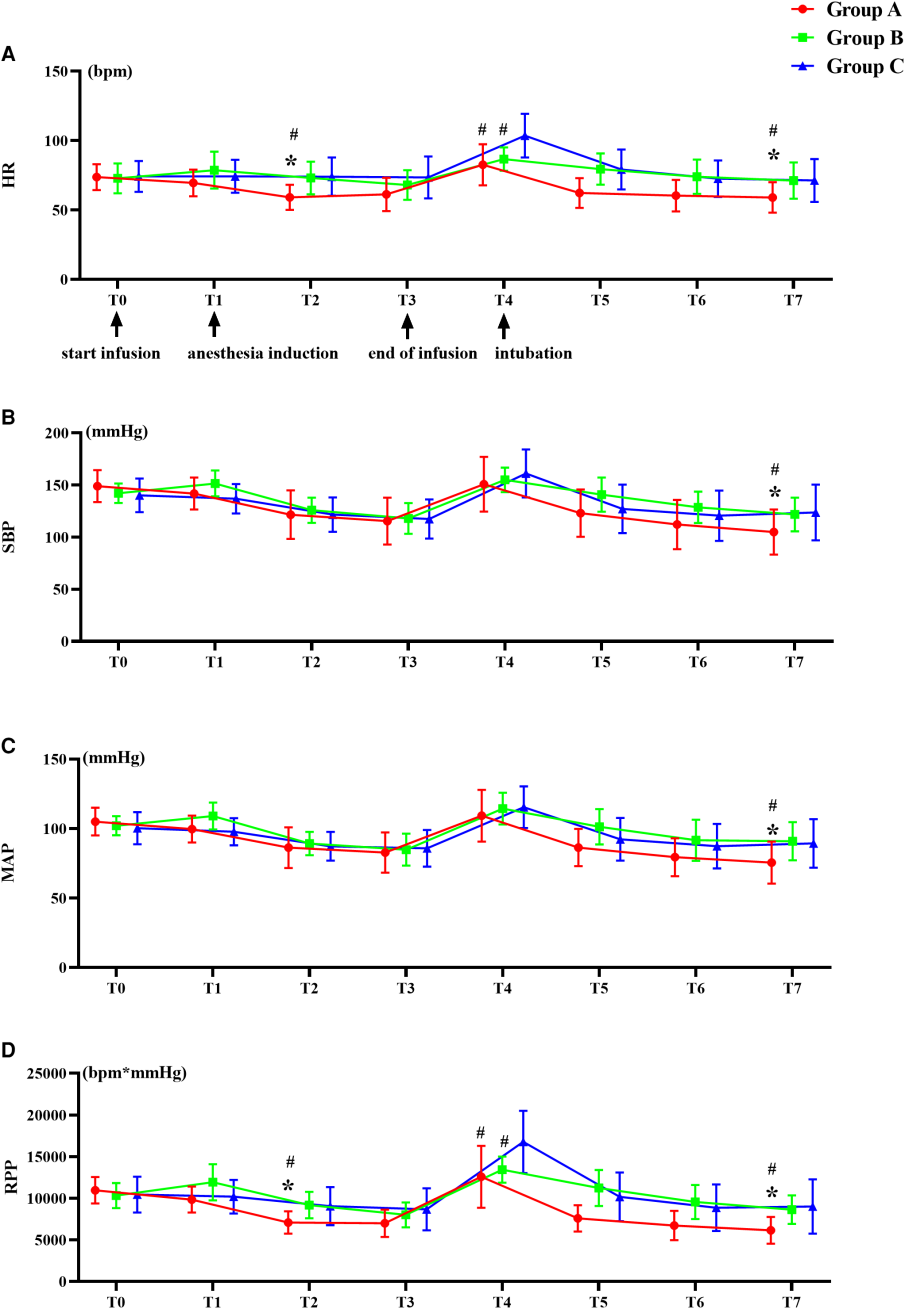
Trends of hemodynamic changes. (**A**) Heart rate (HR), (**B**) Systolic blood pressure (SBP), (**C**) Mean blood pressure (mAP), and (**D**) Rate pressure products (RPP). **^#^***P *< 0.05 versus group C, *P < 0.05 versus group B. Data are showed as mean with SD.

The hemodynamic parameters were recorded at baseline (T0), 5 min after the infusion of the drug (T1), at anesthesia induction (T2), before intubation (T3) and at 1 (T4), 3 (T5), 5 (T6) and 7 (T7) minutes after intubation with DLT (Figure 2). An increase in mAP or HR by a value of >30% from baseline at any of the observed time points was taken as indicative of an intubation response. The primary outcome of this study was the incidence of the intubation response. The secondary outcomes were changes in hemodynamic and cardiac function during induction of anesthesia and intubation of DLT.

### 2.5. Sample size

We performed a pilot study using modified Dixon's up-and-down method to explore the dose of dexmedetomidine. This pilot study was conducted in 20 patients. The first patient in this trial was randomly selected and received 0.5 µg/kg of dexmedetomidine, with a 0.1 µg/kg dose interval. The outcome was the incidence of the stress response (SR). If the SR failed to attenuate, the dose was increased by 0.1 µg/kg, vice versa, if the outcome was satisfactory, the dose was decreased by 0.1 µg/kg. The results of this test show that the ED50 of dexmedetomidine as an adjuvant to induction was 0.776 µg/kg for intubation of DLT. Thus, we selected 0.8 µg/kg as the dose for the dexmedetomidine group.

Based on previous studies (13–15) and our pilot study, to detect a 45% change in incidence of intubation response, defined as increase in HR or mAP by value of >30% of the baseline value, at a power of 90% and an alpha error of 5%, a total of 23 patients were required in each group. Accounting for a dropout rate of 10%, 26 patients were included in each group.

### 2.6. Statistical analysis

Data were analyzed using the statistical analysis software SPSS 25.0. All quantitative data were tested for normality (using the Shapiro–Wilk test) and presented as mean ± SD when they met the normal distribution and median (range) for those that did not meet the normal distribution. One-way ANOVA test was used for those quantitative parameters that met normal distribution and homogeneity of variance (using Levene test), and nonparametric comparisons (Kruskal–Wallis test) were used for variables that did not meet the normal distribution. *Post hoc* comparisons were conducted using the Bonferroni adjustment. For pairwise comparisons, Bonferroni-adjusted *P*-value was presented. Two-way repeated-measures ANOVA with Bonferroni *post hoc* comparisons analyzed hemodynamic data and cardiac function, and Greenhouse-Geisser results were used if assumption of sphericity was not met (Mauchlys Sphericity Test: *P *< 0.05). Qualitative data were expressed as *n* (%), and comparisons were made using Pearson's chi-square test or Fisher's exact test. *P* < 0.05 was considered statistically significant.

## 3. Results

Figures 1, 2 show the flow diagram for this study, in which 78 patients were assessed for eligibility and 78 adult patients were included. The results of 78 adult patients were analyzed.

All three groups were statistically similar with respect to demographic characteristics, as well as baseline heart rate, blood pressure, and TTE parameters (*P *> 0.05) (Table 1). The quantity of etomidate required for induction was statistically similar between the three groups (group A: 22 ± 4 mg, group B: 23 ± 4 mg, group C: 24 ± 5 mg) (*P* > 0.05).

**Table 1:** Demographics of patients enrolled in the study.

Variable	Group A	Group B	Group C	Overall significance (*P*-value)
	(*n* = 26)	(*n* = 26)	(*n* = 26)	
Age (year)	59 ± 9	55 ± 11	58 ± 10	0.340
Sex				0.698
Male	12 (46%)	13 (51%)	15 (58%)	
Female	14 (54%)	13 (51%)	11 (42%)	
BMI (kg/m^2^)	23 ± 3	23 ± 3	23 ± 3	0.494
ASA				0.517
I	3 (12%)	1 (4%)	4 (15%)	
II	23 (89%)	25 (96%)	22 (85%)	
Baseline HR (bpm)	74 ± 9	73 ± 11	74 ± 11	0.889
Baseline mAP (mmHg)	105 ± 10	102 ± 7	100 ± 12	0.206
Chemotherapy or radiotherapy	5 (19%)	3 (12%)	1 (4%)	0.278
Smoking history	11 (42%)	6 (23%)	10 (39%)	0.304
History of hypertension	6 (23%)	8 (31%)	6 (23%)	0.764
Diabetes mellitus	0 (0%)	3 (12%)	2 (8%)	0.360
Baseline ALB (g/L)	45 ± 3	44 ± 3	45 ± 4	0.552
Baseline LVEF (%)	71 ± 7	69 ± 6	68 ± 7	0.208
Baseline SV (ml)	71 ± 19	73 ± 20	73 ± 21	0.925
Baseline CO (L/min)	5 ± 1	5 ± 1	5 ± 2	0.774
Duration of surgery (min)	126 (65–425)	115 (56–302)	108 (44–309)	0.242
Hospital stay (days)	7 (2–18)	7 (3–10)	6 (4–11)	0.419

Values are presented as mean ± SD, median (range) or *n* (%). (*P < *0.05 vs. Group B, Group C.).

BMI, body mass index; ASA, American Society of Anesthesiologists; HR, heart rate; mAP, mean artery pressure; ALB, albumin; LVEF, left ventricular ejection fraction; SV, stroke volume; CO, cardiac output.

The time taken for laryngoscopy and intubation was statistically similar between the three groups (*P* > 0.05). The incidence of the response of cardiovascular stress to laryngoscopy and DLT intubation was 23.1%, 30.8%, and 65.4% in groups A, B, and C, respectively. There was a significant decrease in the intubation response in groups A and B in comparison with group C (*P* < 0.01); however, there was no significant difference between group A and group B (*P *> 0.05) (Table 2).

**Table 2:** Cardiovascular events.

Variable	Group A	Group B	Group C	Overall significance (*P*-value)
	(*n* = 26)	(*n* = 26)	(*n* = 26)	
SR	6 (23.1%)^†^	8 (30.8%)^†^	17 (65.4%)	0.004
Duration time of SR (min)	0.0 (0.0 – 1.0)^†^	0.0 (0.0 – 5.0)^†^	1.0 (0.0 – 7.0)	0.006
Bradycardia	9 (34.6%)*	0 (0.0%)	3 (11.5%)	0.002
Hypotension	11 (42.3%)*	2 (7.7%)	5 (19.2%)	0.011
Hypertension	2 (7.7%)	2 (7.7%)	4 (15.4%)	0.719
Tachycardia	5 (19.2%)^†^	5 (19.2%)^†^	15 (57.7%)	0.003
RPP > 20,000	1 (3.8%)	0 (0.0%)^†^	6 (23.1%)	0.014
Hypotension during operation	7 (26.9%)	8 (30.8%)	8 (30.8%)	0.940
Receiving vasopressor	9 (34.6%)^†^*	1 (3.8%)	1 (3.8%)	0.002
Receiving atropine	4 (15.4%)	1 (3.8%)	2 (7.7%)	0.489

Values are presented as median (range) or *n* (%). *P* < 0.05 vs. *Group B, ^†^Group C. SR defined as mAP or HR >30% of baseline value. Hypotension defined as mAP <30% of baseline value. Hypertension defined as mAP <30% of baseline value. Bradycardia defined as HR <30% of baseline value or 50 bpm. Tachycardia defined as HR >30% of baseline value.

SR, stress response; RPP, rate pressure products.

After infusion of the drugs and induction of anesthesia, there was a significant decrease in HR and RPP in group A compared with group B and C (Figure 2). In group C, the HR, and RPP at 1 min after intubation increased significantly than group A and B (*P *< 0.01) (Figure 2).

**Figure 3:**
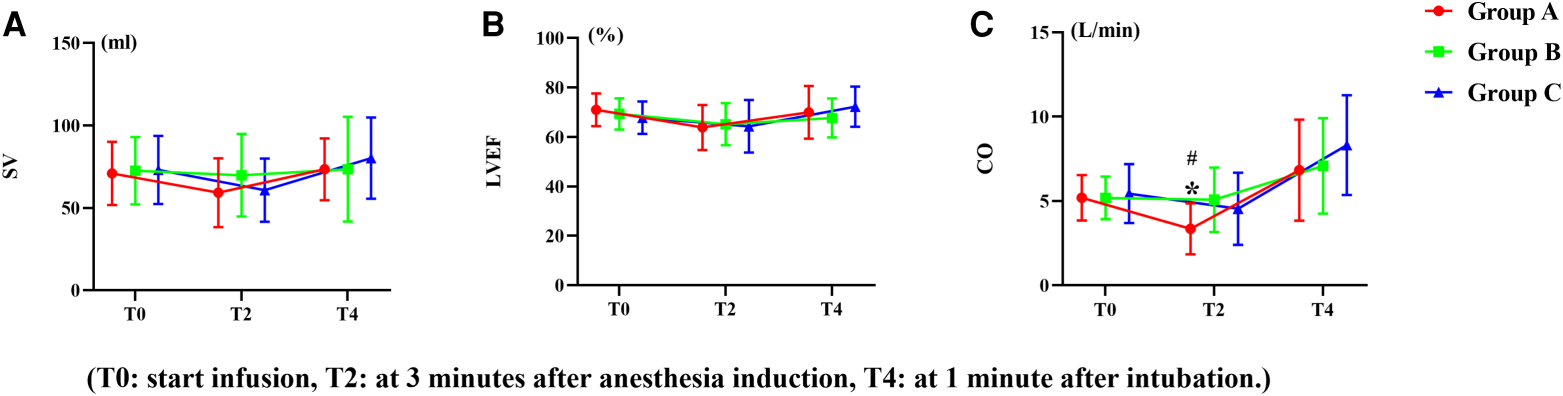
Trends of left ventricular function. (**A**) Left ventricular ejection fraction (LVEF), (**B**) Stroke volume (SV), (**C**) Cardiac output (CO). **^#^***P *< 0.05 versus group C, *P < 0.05 versus group B. Data are showed as mean with SD).

Compared to baseline, LVEF, SV, and CO decreased significantly in the three groups after the infusion of studied drugs and the induction of anesthesia, and CO was significantly lower in group A than in group B and C (Figure 3). There were no significant differences in LVEF, SV, or CO among three groups after intubation (Figure 3).

There were no significant differences in dizziness, hypertension, and tachycardia during recovery form anesthesia among the three groups (Table 3). In addition, the postoperative extubation time was statistically similar between the three groups (group A: 20(10–81) min, group B: 24.5(5–55) min, group C: 27(10–50) min) (*P *> 0.05).

**Table 3:** Postoperative side effects.

Variable	Group A	Group B	Group C	Overall significance (*P*-value)
	(*n* = 26)	(*n* = 26)	(*n* = 26)	
PONV	1 (4.0%)	0 (0.0%)	0 (0.0%)	1.000
Hypertension	3 (11.5%)	3 (11.5%)	6 (23.1%)	0.570
Tachycardia	7 (26.9%)	3 (11.5%)	7 (26.9%)	0.300
Dizziness	1 (3.8%)	4 (15.4%)	3 (11.5%)	0.517
Respiratory failure	1 (3.8%)	0 (0.0%)	0 (0.0%)	1.000
Atrial fibrillation	0 (0.0%)	1 (3.8%)	0 (0.0%)	1.000

Values are presented as number of patients [*n* (%)]. *P* < 0.05 vs. Group B, Group C.

PONV, postoperative nausea and vomiting.

## 4. Discussion

Many studies have evaluated attenuation of the intubation response; however, little attention has focused on the response to cardiovascular stress related to DLT intubation. The results of the present study show that the preinduction administration of a single dose of 0.5 mg/kg esketamine or 0.8 µg/kg dexmedetomidine intravenously resulted in a significant attenuation of the DLT intubation-related cardiovascular stress response during induction of etomidate-sufentanil.

The hemodynamic changes to laryngoscopy and intubation cause HR, BP, and an increase in myocardial oxygen consumption. Previous studies have defined the intubation response as an increase of >20% in HR and/or arterial blood pressure and compared its incidence to evaluate the efficacy of the drugs (16, 17). In our study, we selected patients who required DLT intubation, which could trigger a more intense sympathetic response than SLT intubation; therefore, it is important to maintain hemodynamic parameters within 30% of the individual patient's baseline values (18). So, the primary outcome measure of our study was the incidence of an increase of >30% in HR or mAP. The observation period for the occurrence of the intubation response was taken as 10 min in our study. Compared with previously published data (19), our results suggest that there is a higher intubation response in DLT intubation than in SLT intubation. We think this is due to the characteristics of the DLT (longer length, thicker outer diameter, and stiffer texture of the tube) and the prolonged duration of the DLT insertion time with 55 s in our study vs. the SLT insertion time with 14 s in the previous studies (2, 20).

Esketamine, an optical isomer of ketamine and its analgesic and sedative properties acting on NMDA and opioid receptors, has been used in adjuvant with propofol induction in a dose of 0.5 mg/kg for the obtunding hemodynamic response (21, 22). Based on the previous study (13), we selected esketamine in a dose of 0.5 mg/kg for our study. The dose of dexmedetomidine used for the obtunding intubation response varied from 0.5 µg/kg to 2 µg/kg in previously published studies (16, 17, 23). However, in those studies, dexmedetomidine was the adjuvant with propofol induction rather than etomidate induction. In our pilot study using the modified Dixon method of ascending and falling, an ED50 of dexmedetomidine of 0.776 µg/kg could attenuate the SR of the DLT intubation when it was adjuvant to the induction of etomidate-sufentanil. Therefore, 0.8 µg/kg of dexmedetomidine was chosen in the present study. Unlike previous studies, which only evaluated the effects of the alpha-2 agonists on hemodynamic consequences associated with intubation, our study was first to evaluate the effect of NMDA agonists on intubation response.

In the present study, in the dexmedetomidine group, the HR was significantly lower than in the normal saline group. Many studies have reported that dexmedetomidine could reduce stress hormone levels to control overactivation of the sympathetic system and thus reduces HR (24, 25). As the HR reduced, the RPP (rate pressure product), which represents myocardial oxygen consumption, decreased accordingly, therefore the myocardial oxygen consumption decreased when dexmedetomidine was used as an adjuvant for induction of anesthesia. In the esketamine group, HR and RPP were also lower than normal saline group. We think this is due to its analgesic and sedative effect. Previous studies have shown that esketamine might lead to a better match between myocardial oxygen supply and demand when it used as an adjuvant for induction of anesthesia, and thus reduced cardiac events (5). Our results are similar to those of these studies. In this study, we used TTE to further investigate the direct effects of esketamine or dexmedetomidine on SV, CO, and left ventricular ejection fraction (LVEF). There were no significantly differences in SV or LVEF between the three groups during anesthesia induction. CO in the dexmedetomidine group decreased significantly compared with the esketamine and control group during anesthesia induction. We believe this is due to the decrease in HR caused by the dexmedetomidine, therefore, CO is reduced. During DLT insertion, after a sudden stress response, CO, SV, and LVEF increased among three groups when compared with baseline values; however, the rate of change in CO, SV, and LVEF did not show significant differences between the three groups. These results suggest that both dexmedetomidine and esketamine could better maintain hemodynamic stability and may not directly affect cardiac function when used as an anesthesia adjuvant. This result is consistent with a previous study (26).

There are some shortcomings in our study. This trial was not a multicenter study, and could have been biased in patient selection. Second, echocardiographic indices were measured based on the observation of the measurers. Although each data set was the average value of three cardiac cycles, different observers may have contributed to different results. Another limitation was that the patients of our study had normal left ventricular ejection fraction, so, our results might not be applicable to patients with decreased cardiac function before the operation.

## 5. Conclusions

Based on our results, we conclude that intravenous esketamine 0.5 mg/kg or dexmedetomidine 0.8 µg/kg can attenuate the response to cardiovascular stress to laryngoscopy and DLT intubation. As an adjuvant to etomidate induction, these do not impair cardiac function.

## Data Availability

The original contributions presented in the study are included in the article/Supplementary Material, further inquiries can be directed to the corresponding author.
